# Gut Microbiota as a Driver of Inflammation in Nonalcoholic Fatty Liver Disease

**DOI:** 10.1155/2018/9321643

**Published:** 2018-01-31

**Authors:** Stefano Bibbò, Gianluca Ianiro, Maria Pina Dore, Claudia Simonelli, Estelle E. Newton, Giovanni Cammarota

**Affiliations:** ^1^Department of Clinical and Experimental Medicine, University of Sassari, Sassari, Italy; ^2^Department of Gastroenterology, Catholic University, School of Medicine and Surgery, A. Gemelli Hospital, Rome, Italy; ^3^CytoCure LLC, Beverly, MA, USA

## Abstract

The prevalence of nonalcoholic fatty liver disease and the consequent burden of metabolic syndrome have increased in recent years. Although the pathogenesis of nonalcoholic fatty liver disease is not completely understood, it is thought to be the hepatic manifestation of the dysregulation of insulin-dependent pathways leading to insulin resistance and adipose tissue accumulation in the liver. Recently, the gut-liver axis has been proposed as a key player in the pathogenesis of NAFLD, as the passage of bacteria-derived products into the portal circulation could lead to a trigger of innate immunity, which in turn leads to liver inflammation. Additionally, higher prevalence of intestinal dysbiosis, larger production of endogenous ethanol, and higher prevalence of increased intestinal permeability and bacterial translocation were found in patients with liver injury. In this review, we describe the role of intestinal dysbiosis in the activation of the inflammatory cascade in NAFLD.

## 1. Introduction

Nonalcoholic fatty liver disease (NAFLD) is a multifactorial condition resulting from a complex interaction of genetic and environmental factors. The prevalence of the disorder has increased in recent decades, as is the burden of metabolic syndrome [1]. NAFLD was defined as a spectrum of liver conditions whose dominant feature is abnormal hepatic triglyceride accumulation. In the absence of inflammation and hepatocellular damage, this condition is simply defined as steatosis or nonalcoholic fatty liver. In a liver with chronic NAFLD, lobular inflammation and signs of hepatocellular damage may occur. This latter condition is called nonalcoholic steatohepatitis (NASH). The natural history of NASH is not completely understood, but one can assume that NASH predisposes to several complications such as liver fibrosis, cirrhosis, and hepatocellular carcinoma [2, 3]. Moreover, the pathogenesis of this disorder remains largely unknown. The so-called two-hit hypothesis suggests that accumulation of triglyceride in hepatic cells may expose the liver to secondary insults, primarily oxidative stress, resulting in chronic injury. This model focuses on liver autonomous dysfunction leading to NASH [4]. In recent years, NAFLD was proposed as the hepatic feature of metabolic disorders, as insulin resistance and metabolic syndrome are strongly linked to the progression of liver disease. However, other organs including adipose tissue, muscle, and gut may play an important role in the progression of NAFLD [1, 3]. The liver and intestine are tightly linked through the portal circulation; consequently, gut-derived products, mainly microbial components, arrive primarily to the liver with obvious pathogenic implications [5]. The intestine is colonized by an enormous array of microorganisms, defined as the gut microbiota or microbiome, which can be considered a functional organ [6]. The gut microbiota plays a key role in the maintenance of human health, being involved in the development and growth of the immune system and regulation of several metabolic pathways [7–9]. Quantitative and/or qualitative alterations of gut microbiota, in other way defined as dysbiosis, are known to lead to disruption of this homeostasis and, consequently, development of pathology. Disorders associated with the impairment of gut microbiota can include gastrointestinal diseases [10–13], liver diseases [14], and also metabolic disorders such as metabolic syndrome [15] and diabetes [16, 17].

## 2. The Role of Intestinal Dysbiosis

Our understanding of the relationship between gut microbiota and the development of liver disease has been highlighted in both animal and human studies (see Table 1). Small intestinal bacterial overgrowth (SIBO), increased intestinal permeability, and a number of bacterial endotoxins were reported as putative factors for NASH development [18]. In the first observation, the authors hypothesized an important role for SIBO in the occurrence of NASH, but in recent years, the development of metagenomic sequencing technologies has allowed the description of detailed alterations of the gut microbiota, focusing on qualitative dysbiosis rather than quantitative modifications [19–22].

The prevalence of SIBO in patients with NASH has been widely studied. Wigg et al. reported that patients with NASH have a higher prevalence of SIBO compared to controls (50% versus 22%). In addition, higher levels of TNF-alpha compared to control subjects were observed, although intestinal permeability and serum endotoxin levels were similar in the two groups [23]. These findings were only partially confirmed by further studies. Miele et al. found that subjects with NAFLD had significantly increased gut permeability and a higher prevalence of SIBO, compared with healthy subjects. Both gut permeability and the prevalence of SIBO correlated with the severity of steatosis but not with the presence of NASH [24]. These findings were confirmed in a further study by Shanab et al.; additionally, authors found an enhanced expression of Toll-like receptor 4 (TLR4) and release of interleukin 8 (IL-8) [25]. Moreover, increased intestinal permeability and higher levels of blood lipopolysaccharide (LPS) were found in children with NASH compared to those with NAFLD [26]. These data confirmed findings based on rodent models, showing that higher intestinal mucosa permeability promotes the increase of LPS levels in portal blood and in turn liver inflammatory damage [27–29].

The gut microbiota has been suggested to be responsible for the increase of endogenous ethanol production in patients with NAFLD. A rodent experimental model demonstrated an increased breath ethanol content [30] that was abolished by treatment with neomycin. This observation was also confirmed in humans. Patients with NASH harbored an increased number of alcohol-producing bacteria (in particular *Escherichia coli*) in their microbiome associated with elevated blood-ethanol concentration [31]. An additional study confirmed the results [32]. Patients with NAFLD had a prevalence of SIBO of 37.5%, and *Escherichia coli* was the predominant bacteria in duodenal fluid aspirate. Moreover, patients with SIBO had higher endotoxin levels and expression of Toll-like receptor 4 (TLR4) compared to those without [32]. However, the presence of SIBO appears not to represent an ubiquitous marker of NAFLD. A study performed on 20 patients with NAFLD showed intestinal permeability, and alcohol and endotoxin levels in the plasma were significantly higher compared to controls, but the prevalence of SIBO was similar between patients and controls [33].

The development of modern sequencing techniques (metagenomic approach) has allowed a deeper analysis of the microbiota composition [34]. The first metagenomic characterization of gut microbiota in patients with NASH was reported by Mouzaki et al. [35]. The percentage of *Bacteroidetes* and *C. coccoides* was lower in patients with NASH compared to patients with NAFLD and healthy controls. The percentage of *Bacteroidetes* in patients with NASH remained significantly lower even after adjusting for anthropometric variables (body mass index) and fat intake [35]. Boursier et al. were able to partially confirm the data. In their study, patients with NASH harbored a higher quantity of *Bacteroides* and a lower quantity of *Prevotella*, compared to individuals without NASH [36]. The multivariate analysis adjusted for metabolic factors showed that *Bacteroides* abundance was independently associated with NASH. Differences in taxonomic composition of intestinal microbiota at the phylum level according to NAFLD severity were not detected. On the contrary, dramatic differences were observed at the family level according to severity of hepatic injury. More specifically, *Bacteroidaceae* family increased along with severity of liver lesions, whereas the family of *Prevotellaceae* and *Erysipelotrichaceae* decreased. Authors also evaluated the correlation with the grade of liver fibrosis. Patients with a grade of liver fibrosis of F0/F1 had higher abundances of *Bacteroides* and *Ruminococcus* and lower abundance of *Prevotella* compared to those with F2 liver fibrosis [36]. Analysis of the fecal microbiome and volatile organic compound (for instance ethanol) in patients with NASH revealed a significant increase in fecal volatile compounds in NAFLD patients compared to healthy controls. In the microbiome of NAFLD patients, *Lactobacillus* species and selected members of phylum Firmicutes, in particular *Lachnospiraceae* (*Dorea*, *Robinsoniella*, and *Roseburia)*, were overrepresented, while other members (Ruminococcaceae; genus, *Oscillibacter*) were significantly underrepresented [37]. Further data show that patients with NASH have a higher abundance of *Parabacteroides* and *Allisonella* and lower representation of *Faecalibacterium* and *Firmicutes* families [38].

The intestinal dysbiosis is able to modify the profile of bile acids in patients with NAFLD. In a population of patients with NASH, levels of unconjugated cholic acid and chenodeoxycholic acid were, respectively, increased. The analysis of intestinal microbiota revealed that patients with NASH harbored a lower relative abundance of *Bacteroidetes* and *Clostridium leptum*, independently from other metabolic factors [39]. For instance, *Clostridium leptum* is able to modify bile acids, converting them from primary to secondary bile acids [40]. The correlation of bile acid levels and intestinal dysbiosis with markers of hepatic injury suggests a possible role for bile acids in the progression of NAFLD to NASH [39].

In pediatric patients, NAFLD-specific alterations in gut microbiota composition, different from those found in adults, were also described. Children with NASH had an increased number of *Bacteriodetes* and *Proteobacteria* and a decreased number of *Firmicutes* and *Actinobacteria* compared to healthy children [31]. In a more recent research, a similar dysbiosis pattern was observed in pediatric patients characterized by a decrease in *Oscillospira* and *Rikenellaceae* and an increase in *Bradyrhizobium*, *Anaerococcus*, *Peptoniphilus*, *Propionibacterium acnes*, *Dorea*, and *Ruminococcus* [41].

## 3. The Role of Immunity

As discussed before, dysbiosis plays a main role in increasing intestinal permeability, with consequent passage into the portal circulation of bacteria-derived products. Among these, the lypopolisaccharide (LPS), a cell component of Gram-negative bacteria, is the best investigated. LPS is able to activate Toll-like receptors (TLRs) resulting in the production of proinflammatory cytokines and chemokines. Several experimental models of NASH reported high levels of LPS leading to hepatic injury through the recruitment of inflammatory cells [5, 42]. In this pathway, a key role is played by Kupffer cells. They contribute to endotoxin clearance [43] and to inflammatory response, through several TLRs located in their surface that, after being activated by LPS, are able to trigger a cascade of events, leading to the production of inflammatory cytokines, such as IL-1*β* and TNF-*α* [44].

Up to 13 different TLRs have been identified in mammals; however, among these, only TLR2, TLR4, TLR5, TLR6, TLR7, and TLR9 are known to be involved in the pathogenesis of NAFLD [45]. TLR2 mainly binds peptidoglycan and lipoteichoic acid that are components of Gram-positive bacterial cells. In a murine experiment, Miura et al. [45] demonstrated that TLR2-deficient mice are resistant to diet-induced steatohepatitis, showing a lower expression of proinflammatory cytokines (TNF*α* and IL-1*β*) [46]. In contrast, in other experiments on TLR2-deficient mice, a similar and even more severe susceptibility to steatohepatitis was observed [47, 48]. Studies looking at the interaction between TLR2 and TLR6 found that deregulation of TLR6 expression potentiated the TLR2-mediated liver inflammation. Indeed, the TLR2/TLR6 stimulation promoted the production of proinflammatory cytokines that was higher in patients with lobular inflammation [49]. TLR4 is a receptor for LPS. The importance of this axis has been clarified through TLR4 mutant mice resistant to the development of NAFLD [50]; furthermore, a direct link between TLR4 and Kupffer cells was described in the pathogenesis of steatohepatitis, as the experimental destruction of Kupffer cells was shown to prevent the increased expression of TLR4 [51]. The relevance of this interplay was confirmed in a murine model, where occurrence of NAFLD required endotoxin-dependent activation of hepatic Kupffer cells, associated with SIBO and enhanced intestinal permeability [52]. TLR5 is a receptor for bacterial flagellin. Although few data are reported about its role in the development of metabolic disorders, a murine model suggests that TLR5 deficiency is able to promote obesity, steatosis, and in turn metabolic syndrome [53]. More recent evidence shows that hepatocyte TLR5 protects against diet-induced liver disease [54]. Similarly, a protective role in preventing NAFLD was also reported for TLR7 [55]. TLR9 is a receptor for bacterial DNA, in particular for the unmethylated CpG motif, which is increased in NASH models; the activation of TLR9 signaling on Kupffer cells induces the production of proinflammatory cytokine, such as IL-1*β* leading to steatosis and inflammation. Moreover, the activation of TLR9 in hepatic stellate cells suggests a role in promoting fibrogenesis [56]. In animal models, the blockage of Il-1 signaling leads to a reduction of TLR9-mediated liver damage, in particular the endogenous IL-1 receptor antagonist, and regulates the extent of TLR9-induced liver injury [57].

The myeloid differentiation primary response gene 88 (MyD88) is the most investigated signaling adaptor for TLRs. The activation of this adaptor by TLRs, mainly TLR4 and TLR9, results in the upregulation of the transcriptional factor nuclear factor kappa beta (NF-*κ*B) and c-Jun N-terminal kinase (JNK) pathway [58]. However, data about the role of Myd88 in the pathophysiology of NAFLD are conflicting. For example, Duparc et al. recently reported that, in a rodent model, the hepatocyte specific deletion of Myd88 predisposes to inflammation, hepatic steatosis, and insulin resistance [59]; other reports suggested that the deletion of Myd88 increases the risk of developing features of metabolic syndrome such as diabetes and hepatic steatosis [60, 61]; conversely, deletion of MyD88 in intestinal epithelial cell-specific murine model partially protected against diet-induced obesity, diabetes, and metabolic inflammation [62].

Finally, the inflammasome, a group of sensors for endogenous and/or exogenous pathogen-associated molecular patterns (PAMPs) or damage-associated molecular patterns (DAMPs) [63], seems to be involved in development of liver steatosis and inflammation. The inflammasome is a multimeric signaling platform that leads to the production of IL-18 and IL-1 through NRLP3 (NOD-like receptors, pyrin domain containing 3) and NRLP6 (and 6). Interestingly, in inflammasome-deficient mice, an increase of *Bacteroidetes* and a reduction of *Firmicutes* were reported, resulting in a higher activation of TLR4 and TLR9 and subsequent inflammatory pathway [64].

## 4. Concluding Remarks

**Figure 1:**
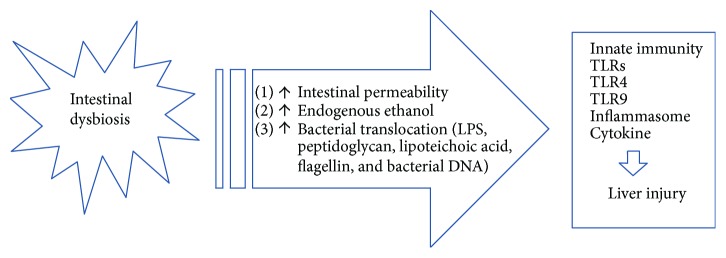
Interactions between gut microbiota and innate immunity in the pathogenesis of steatohepatitis.

Gut microbiota alterations and increased intestinal permeability appear to play a major role in promoting inflammation and progression of NAFLD to NASH. The disruption of “normal” microbiota can occur in several conditions including environmental exposures, medications, or diet [65, 66]. It was hypothesized that intestinal dysbiosis may lead to the progression of NAFLD through several pathways. The presence of SIBO is related to endogenous production of alcohol and furthermore to increased intestinal permeability, favoring the passage of bacterial-derived products in the portal circulation. These products (LPS, peptidoglycan, lipoteichoic acid, flagellin, and bacterial DNA) are ligands for TLRs and stimulate the innate immune system in the liver (Figure 1).

Several TLRs, identified in the liver, have a mandatory role in hepatic injury mechanisms, as reported in some animal studies described specifically in the previous section. It has been described that different bacterial products have a selectivity for TLRs, which have different roles in the progression of tissue inflammation. For example TLR4 and TLR9, which bind LPS and bacterial DNA, respectively, promote inflammation and liver fibrogenesis through the activation of Kuppfer cells and hepatic stellate cells. Conversely, certain receptors for bacterial-derived products may have a protective role in the progression of inflammation; indeed, it has been observed that the specific deletion of TLR5 and TLR7 promotes the inflammatory pathways.

Consequently, the altered balance of these receptors can trigger a cascade of events, in particular the secretion of proinflammatory cytokines that drive the inflammation in NAFLD. This condition, previously described as “metabolic endotoxiemia,” is a common feature of several metabolic disorders [28, 67].

The reported evidences about the inflammatory pathways mainly derive from animal models. These findings are sometimes conflicting; furthermore, they are not always confirmed by “human” studies. For example, the hepatic deletion of MyD88 seems to promote a proinflammatory “milieu,” while the specific deletion in intestinal epithelial cells may have a protective role. There is still much to be understood about the role of the intestine in the inflammatory mechanisms of NAFLD.

In addition, qualitative alterations of gut microbiota are able to interfere with the intestinal absorption of bile acid. Based on this observation, a new role for gut microbiota was proposed. More specifically, intestinal dysbiosis resulting in higher levels of unconjugated bile acid, able to inhibit farnesoid X receptor (FXR) signaling, was observed in animal models [68]. FXR inhibition results in increased production of ceramides that cause lipid toxicity and increased fatty acid synthesis.

In conclusion, to date, evidence for a role of gut microbiota in the progression of NAFLD is still weak, although the reported observations are very intriguing [69]. Research fields that need to be explored are many, from the identification of specific alterations of the gut microbiota, to a more detailed understanding of the mechanisms of innate immunity. The comprehension of the pathogenic pathways of NAFLD in lean patients is a very interesting issue, and several evidences suggest a main role for gut microbiota [70].

These observations allow us to consider a new role for the intestine, suggesting it as one of the main actors in NAFLD/NASH progression. Indeed, the altered production of volatile metabolites by gut microbiota, such as endogenous alcohol, and the uncontrolled passage of bacterial-derived products in the bloodstream would be able to trigger the inflammation and cellular damage in the liver, even in subjects without overt metabolic syndrome. Assuming these data, the gut can be placed side by side with muscle and adipose tissue as a “director” in the progression of liver disease; it would be very important to understand whether intestinal dysbiosis is a factor necessary for the development of NASH, or is only a precipitating factor.

Finally, further studies are needed, and maybe in a future not too far, they will provide new therapeutic chances for this disorder of growing worldwide interest.

## Figures and Tables

**Table 1:** Gut microbiota alteration in human studies (NASH = nonalcoholic steatohepatitis; SS = simple steatosis; HC = healthy controls).

Study	Subjects	Gut microbiota alterations
Mouzaki et al. [35]	NAFLD (SS or NASH) and HC	↑ *Clostridium coccoides* in NASH versus SS ↓ *Bacteroidetes* in NASH versus SS and HC
Boursier et al. [36]	NAFLD (SS, NASH, and fibrosis)	↑ *Bacteroides* and ↓ *Prevotella* in NASH ↑ *Bacteroidaceae*; ↓ *Prevotellaceae* and *Erysipelotrichaceae* according to the severity of NASH ↑ *Bacteroides* and *Ruminococcus* and ↓ *Prevotella* in patients with F2 fibrosis versus F0/F1
Raman et al. [37]	NAFLD and HC	↑ *Lactobacillus* and selected members of *Firmicutes* (*Dorea*, *Robinsoniella*, and *Roseburia*); ↓ one member of *Firmicutes* (*Oscillibacter*) in NAFLD
Wong et al. [38]	NASH and HC	↑ *Parabacteroides* and *Allisonella*; ↓ *Firmicutes* and *Faecalibacterium* in NASH
Mouzaki et al. [39]	NAFLD (SS and NASH) and HC	↓ *Bacteroidetes* and *Clostridium leptum* in NASH versus HC
Zhu et al. [31]	Children—NASH, obese, and HC	↑ *Bacteriodetes* and *Proteobacteria* and ↓ *Firmicutes* and *Actinobacteria* in NASH versus HC
Del Chierico et al. [41]	Children—NAFLD (SS and NASH), obese, and HC	↑ *Bradyrhizobium*, *Anaerococcus*, *Peptoniphilus*, *Propionibacterium acnes*, *Dorea*, and *Ruminococcus* and ↓ *Oscillospira* and *Rikenellaceae* in NAFLD
